# Effect of Massage Therapy for the Treatment of Neonatal Jaundice: A Systematic Review and Dose-Response Meta-analysis

**DOI:** 10.1155/2022/9161074

**Published:** 2022-03-20

**Authors:** Marjan Shahbazi, Salman Khazaei, Samad Moslehi, Fatemeh Shahbazi

**Affiliations:** ^1^Department of Occupational Therapy, School of Rehabilitation, Shahid Beheshti University of Medical Sciences, Tehran, Iran; ^2^Department of Epidemiology, School of Public Health, Hamadan University of Medical Sciences, Hamadan, Iran; ^3^Research Center for Health Sciences, Hamadan University of Medical Sciences, Hamadan, Iran; ^4^Department of Biostatistics, School of Public Health, Hamadan University of Medical Sciences, Hamadan, Iran; ^5^Students Research Committee, Hamadan University of Medical Sciences, Hamadan, Iran

## Abstract

**Background:**

The effectiveness of massage therapy in the treatment of neonatal jaundice has been established in previous literature, but how much the level of massage can reduce the mean of bilirubin in neonates with jaundice is a question that has been addressed in this review.

**Methods:**

Four electronic databases, including Cochrane, PubMed, Scopus, and Web of Science, were searched for relevant literature. For the dose-response association between massage therapy and treatment of neonatal icterus, we conducted a meta-analysis using the random-effects model. For any level of intervention, we calculated the overall mean difference (MD) with 95% confidence intervals (CI).

**Results:**

Twenty studies were included in our meta-analysis. There was a positive and significant increasing dose-response trend between massage therapy and the mean reduction of bilirubin in neonates with hyperbilirubinemia as follows: <50 minutes massage during the experiment -0.36 (95% CI: -0.67, -0.06; *I*^2^ = 66%), 50-60 minutes massage during the experiment -0.41 (95% CI: -0.95, 0.13; *I*^2^ = 84%), and ≥101 minutes massage during the experiment -1.20 (95% CI: -1.63, -0.78; *I*^2^ = 83%). The heterogeneity across studies was mild to moderate.

**Conclusions:**

The presence of a dose-response relationship favors the causal relationship between massage therapy and reduction of neonatal jaundice.

## 1. Introduction

Neonatal jaundice is a common and usually harmless condition in newborn babies that make yellowing of the skin and the whites of the eyes [1]. This disorder is reported in more than half of newborns and 80% of preterm children [2]. There are several risk factors for it, but the main ones are premature birth, different blood types of mother and baby, babies with East Asian ancestry, and breastfeeding [3–9]. Jaundice can cause acute bilirubin encephalopathy and kernicterus in severe cases [10, 11].

Several treatment approaches have been described for neonatal hyperbilirubinemia and include phototherapy, enhanced nutrition, intravenous immunoglobulin, and neonatal exchange transfusion [12–14]. In recent years, massage therapy has been introduced as a new method in the treatment and care of neonates with jaundice [15]. Massage can also promote physical and intellectual improvement, immunity, bone mineral density, sleep, digestion and absorption, and emotional connection between mothers and infants [16–20].

Currently, the effect of massage on neonatal growth and health care is well established [21–24]. But, there is no general agreement about the effect of massage on neonatal jaundice. In this situation, meta-analyses are one of the best methods for scientifically accumulating all the available evidence and can offer the best and most reliable sources of evidence. Based on the latest meta-analysis conducted by Zhang et al. in 2018, massage therapy can significantly decrease serum and transcutaneous bilirubin levels [25]. In this updated systematic review, we aimed to assess the dose-response relationship between massage therapy and neonatal jaundice. Unlike the previous meta-analysis, for the first time, we investigated the nonlinear effect of the massage as a continuous variable in our models with the spline method.

## 2. Methods

### 2.1. Search Strategy

In this review, four electronic databases including PubMed, Scopus, Web of Science, and Cochrane Library were searched for the relevant studies published on October 2021. The search start date was not limited. We searched different combinations of keywords and MeSH terms (((((*massage*) OR (*physiotherapy*)) OR (“*Physical Therapy Modalities*”)) OR (“*physical therapy*”)) OR (“*touch therapy*”)) AND ((((*Jaundice*) OR (*Bilirubin*)) OR (*Hyperbilirubinemia*)) OR (*icterus*)). Duplicate reports of the same study were deleted. If the full text of the paper was unavailable, we received the paper from Sci-Hub; otherwise, we requested the article from the author via email. We also scanned the reference lists of all included studies and pertinent reviews for additional references.

### 2.2. Study Selection

This study was conducted based on the criteria of the PRISMA guidelines. In this systematic review and dose-response meta-analysis, the published parallel randomized clinical trials (RCTs) or controlled clinical trials (CCTs) for the treatment of neonatal icterus were included, regardless of the language, geographical location, date of publication, nationality, race, and gender. Other study designs, such as case reports, case series, letters to the editor, observational studies (including case-control, cohort, and cross-sectional studies), and narrative reviews, were excluded. The study population was babies within 28 days of delivery that received massage therapy to reduce bilirubin level (in other words, neonates in the intervention group received massage therapy in addition to routine therapy). The control group included neonates who underwent routine comprehensive treatment such as phototherapy and immunoglobulin supportive treatment. We excluded studies that newborns with jaundice suffer from other serious illnesses, such as severe respiratory distress syndrome and severe congenital heart disease. When the results of the same study were published in more than one paper, only the most recent and/or complete article was included in the analysis.

### 2.3. Screening and Data Extraction

All potentially relevant publications were inserted in EndNote X8 software and reviewed independently by two authors (F.S. and M.S.). Discrepancies between authors were resolved by consensus with an expert (S.KH.). The two authors extracted the data to identify eligible studies. After the final evaluation, the authors extracted and recorded the following data: name of the first author, date of publication, country, number of a participants in each arm of the RCT, study design, the mean and standard deviation (SD) of transcutaneous or serum level of bilirubin in each intervention group, trial duration, duration of massage therapy (minutes in each session), and frequency (the number of massage sessions in each day). We also contact the corresponding author to obtain the data when necessary. Then, qualified studies were obtained for full-text screening. For this study, the primary outcomes are the serum bilirubin level and transcutaneous bilirubin level on each day of the intervention. For this study, we defined three levels of massage therapy including ≤50 minutes during the experiment, 51 to 100 minutes during the experiment, and ≥101 minutes during the experiment. In all studies, baseline measure before starting the experiment was considered as the reference group.

### 2.4. Quality Assessment of Studies

To assess the methodological quality of included studies, we used the Cochrane criteria for RCT studies. We assessed the quality of all relevant studies based on the random sequence generation, allocation concealment, blinding of participants and personnel, blinding of outcome assessment, incomplete outcome data, and selective reporting. Then, the included RCTs were classified to low risk of bias if all mentioned criteria were met, unclear risk bias if one item were not met, and high risk of bias if more than one item were not met.

### 2.5. Statistical Analysis

We extracted the mean and standard deviation of serum bilirubin level and transcutaneous bilirubin in each arm of RCT measured based on the milligram per deciliter (mg/dl) and then calculated the mean difference (MD), standard mean difference (SMD), and standard error for the mean difference. In some studies, instead of mean and SD for bilirubin level, the median, 25^th^, and 75^th^ percentiles were reported that these measures convert to equivalent mean and SD using the following formulas:

(1) $$\begin{array}{c} \text{Mean}=\frac{\sum \left(25^{\text{th}}+50^{\text{th}}+75^{\text{th}}\text{percentiles}\right)}{3}\\ \text{SD}=\frac{75^{\text{th}}\text{percentile}-25^{\text{th}}\text{percentile}}{3} \end{array}$$

The chi-square (chi^2^) test of heterogeneity was performed to detect heterogeneity among the studies. The *I*^2^ statistic was applied to determine the degree of heterogeneity between the studies. The random-effects model was applied to produce the pooled estimates. The pooled associations with 95% confidence intervals (CI) were presented separately for each level of massage therapy. Moreover, using the “drmeta” command in Stata software, we conducted dose-response meta-analysis to evaluate the relationship between massage therapy (minutes per day) and mean of bilirubin level (mg/dl). This approach was based on the methods described by Orsini et al. for ordinal data to generate dose-response curves [26]. In this method, two-stage random-effects models (linear and cubic spline) were performed, and the best model was chosen according to the Akaike information criteria and Bayesian information criteria [27]. For the dose-response meta-analysis, the total duration of the massage per day was set as the midpoint in each category. To drive the dose-response curve, the duration of massage therapy was modeled using restricted cubic splines with three knots in fixed percentiles (25%, 50%, and 75%) of the distribution. We performed a two-stage random-effects dose-response meta-analysis to examine the nonlinear relationship between massage therapy and mean of bilirubin. In the first stage, a restricted cubic spline model with 2 spline transformations (three knots minus one) was fitted taking into account the correlation within each set of published relative risks. In the second stage, we combined the two regression coefficients and the variance/covariance matrices that had been estimated within each study, using the multivariate extension of the method of moments in a multivariate random-effects meta-analysis. We used the funnel plot, Begg [28], and Egger [29] tests and the “trim and fill” method [30] to examine the possibility of publication bias. The analysis was performed using both Review Manager 5.4 software and Stata 16 software (StataCorp, College Station, TX, USA).

## 3. Results

### 3.1. Description of Studies

**Figure 1:**
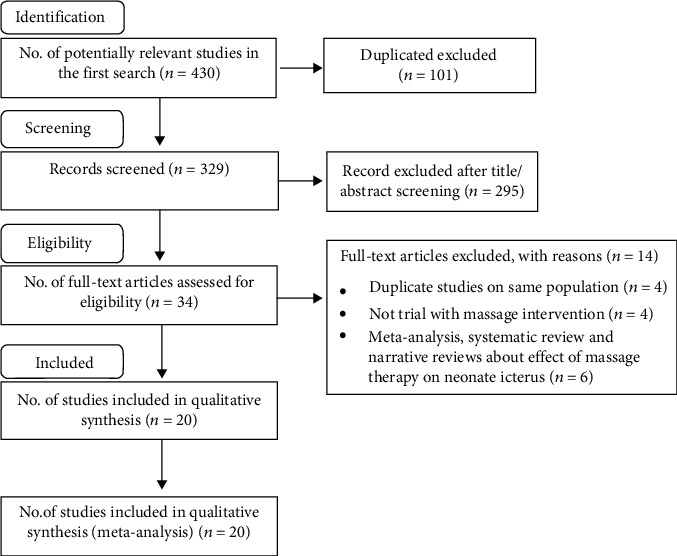
Flow diagram of the study selection process including publications for the dose-response meta-analysis of massage therapy and neonate jaundice.

The Preferred Reporting Items for Systematic Reviews and Meta-Analyses (PRISMA) literature search flowchart is presented in Figure 1. We retrieved 430 publication titles, and after the title and abstract screening, evaluation of 34 full-text reports resulted in the identification of 20 trial articles that could be included in the dose-response meta-analysis. Reasons for exclusion included duplicate reports on the same population (*n* = 6); not a trial with massage intervention (*n* = 11); and meta-analysis, systematic review, and narrative reviews about the effect of massage therapy on neonatal icterus (*n* = 8). We investigated 607 neonates in the control group and 588 cases in the intervention group. The studies had been published in 4 countries. All studies reported adjusted effect sizes. The characteristics of the included studies are given in Table 1.

### 3.2. Dose-Response Association

**Figure 2:**
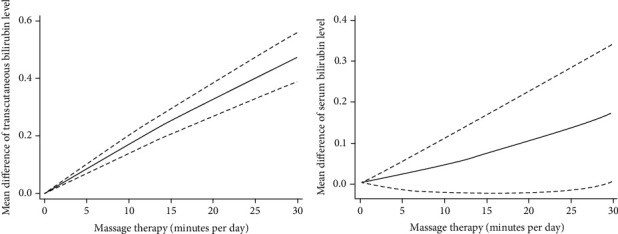
The dose-response relationship between massage therapy (per minute in day) and mean of serum/transcutaneous bilirubin level (mg/dl); the solid line represents the fitted linear trend, and the dashed line represents the 95% confidence interval.

Using “drmeta” command, there was a significant linear association between massage therapy and mean reduction in transcutaneous/serum bilirubin levels [mean difference and 95% CI for transcutaneous bilirubin level: 1.75 mg/dl (1.42, 2.08); mean difference and 95% CI for serum bilirubin level: 0.47 mg/dl (0.18, 1.07)] (Figure 2). Actually, for each minute's increase in massage therapy, the mean difference of transcutaneous and serum bilirubin levels increases 1.75 mg/dl and 0.47 mg/dl, respectively.

### 3.3. Effect of Massage Therapy on the Treatment of Neonatal Jaundice

**Figure 3:**
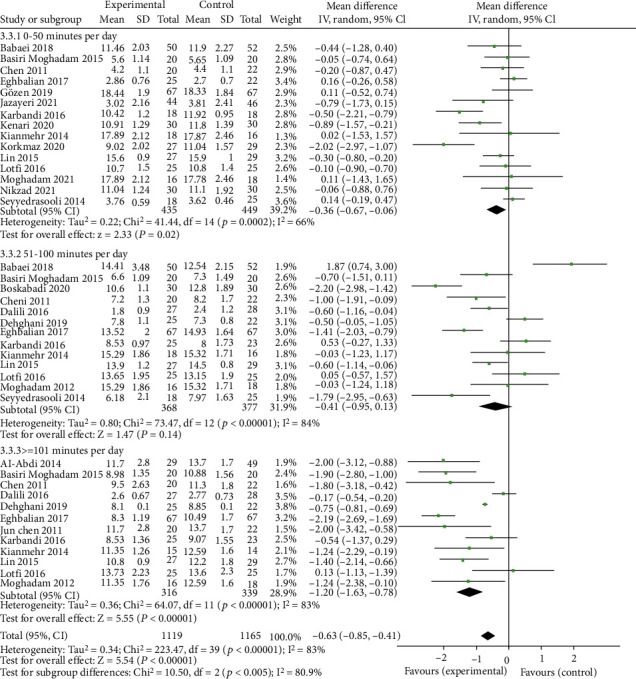
Forest plot of the association between massage therapy and neonatal jaundice in each category of massage therapy.

As shown in Figure 3, there was a significantly increasing dose-response trend between massage therapy and the mean difference of bilirubin level. As the level of intervention with massage increases, the mean difference between the intervention and control groups also increases. In other words, the mean bilirubin level in the intervention group with ≤50 minutes of massage during the trial was 0.36 mg/dl lower than the control group. Also, the mean of bilirubin level in second and third categories of intervention who received 51-100 and ≥101 minutes of massage during the study was 0.41 and 1.20 mg/dl lower than the control group. The heterogeneity between the included studies was moderate in all levels of intervention.

### 3.4. Publication Bias

The Begg and Egger tests revealed no evidence of publication bias (*P* = 0.194 for the Begg test and *P* = 0.156 for the Egger test). Also, trim and fill analysis estimated no missing studies and no evidence of publication bias.

### 3.5. Quality Assessment of Included RCTs

**Figure 4:**
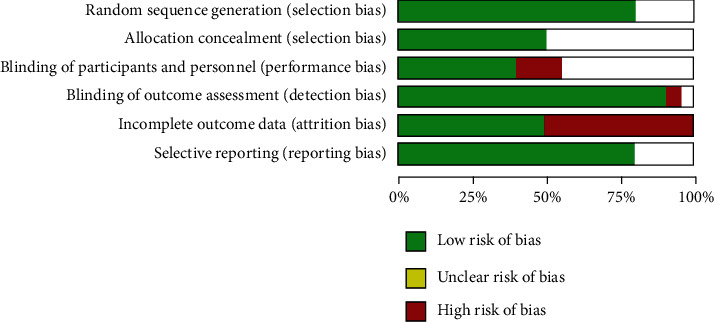
Risk of bias graph for included studies in dose-response meta-analysis.

The risk of bias graph for the selected studies in our dose-response meta-analysis generated by RevMan 5.3 is shown in Figure 4. Random sequence generation was observed in more than 75% of studies. The condition of allocation concealment was unclear in almost half of the cases and observed in remaining 50 percent. In more than 80% of the studies, the outcome assessment situation was well blinded. More details about other errors are shown in Figure 4.

## 4. Discussion

The result of this dose-response meta-analysis revealed that an increase in the amount of massage therapy per minute is significantly associated with decreased neonatal jaundice. This evidence shows a dose-response relationship between massage therapy and neonatal jaundice. When a dose-response relationship is present, it adds plausibility to a causal relationship between exposure and outcome [31].

The result of the present study indicated that the level of bilirubin in the neonate with jaundice decreases as the level of intervention to massage increases. Based on the previous RCTs and CCTs, the mean of total bilirubin levels in intervention groups who receive massage and control groups were similar at baseline and before any intervention. However, with increasing the frequency and duration of massage therapy, its effectiveness increases significantly. In justifying this issue, we can say massage increases lymph flow and blood circulation [32]. This increase in blood circulation can cause bilirubin isomers to be excreted more rapidly into the feces. Additionally, massage facilitates the release of meconium and reduces the reabsorption of bilirubin into the blood. Stools contain large amounts of bilirubin, which delay excretion associated with increased bilirubin levels. Therefore, an increase in bowel movements through massage therapy can increase bilirubin excretion [33].

The inclusive studies in this meta-analysis were related to 4 countries: Iran, Japan, China, and Turkey. These countries are located in Asia. Based on the previous research, the Asian ethnicity increases the chance of neonatal jaundice. Also, the American Academy of Pediatrics clinical practice guideline for the management of newborn hyperbilirubinemia reported that Asian race is a risk factor for the development of newborn hyperbilirubinemia. Differences in the risk of hyperbilirubinemia likely relate to the distribution of gene polymorphisms that are associated with hyperbilirubinemia. For example, mutations of the UGT1A1 gene are linked to an increased risk of neonatal hyperbilirubinemia in Asian populations, as compared with white populations. In the absence of genetic testing, race or ethnicity is likely a rough approximation of the likelihood of these genetic variations related to hyperbilirubinemia [9, 34].

There were a few limitations and potential biases in this meta-analysis. First, some studies seemed potentially eligible to be included in our meta-analysis, but we could not access their full text. This issue may raise the possibility of selection bias. Second, in randomized clinical trial studies included in our meta-analysis, some participants had dropped out due to the length of follow-up. It might introduce selection bias in our results. Third, different methods of massage therapy in included RCTs may affect the pooled results.

Additionally, examining the dose-response relationship made it possible to know how much intervention to massage reduced the mean of bilirubin in babies with jaundice. Furthermore, a wide search strategy in this research increased the sensitivity of the search to include as many relevant articles as possible.

## 5. Conclusion

The results of this meta-analysis suggest that the mean of bilirubin decreases as the level of intervention with massage (in terms of duration and frequency per minute) increases. This finding confirms the linear relationship between massage therapy and the treatment of neonatal jaundice. The existence of a dose-response relationship between massage therapy and the mean of bilirubin in neonates with hyperbilirubinemia can strengthen the scientific background for therapeutic intervention in the newborn intensive care unit (NICU) for the treatment of neonatal jaundice. Finally, it seems that using massage therapy can help prevention of hospitalization in neonate with jaundice, reduce the length of hospitalization, or prevent exchange transfusions.

## Figures and Tables

**Table 1:** Description of eligible studies reporting the effect of massage therapy on neonatal jaundice.

First author, year	Country	Sample size (I/C)	Gestational age (week)	Apgar score	Birth weight (g)	Intervention characteristics
Al-Abdi, 2014 [35]	Iran	29/49	37-41	8-10	2800-3600	10 days, one time in each day, 15-20 minutes in each time.
Dalili, 2016 [36]	Iran	27/28	37-41	8-10	2800-3600	4 days, three times in each day, 15-20 minutes in each time.
Babaei, 2018 [37]	Iran	50/52	37-41	8-10	≥2500	4 days, one time in each day, 15-20 minutes in each time.
Basiri-Moghadam, 2015 [38]	Iran	20/20	34-36	8-10	1500-2500	4 days, two times in each day, 20 minutes in each time.
Dehghani, 2019 [39]	Iran	25/22	37-42	8-10	≥2500	7 days, two times in each day, 15 minutes in each time.
Eghbalian, 2017 [40]	Iran	67/67	38-40	8-10	No. reported	4 days, two times in each day, 15-20 minutes in each time.
Jazayeri, 2021 [41]	Iran	18/18	≥35	No. reported	≥2500	1 day, once in each day, 15 minutes in each time.
Karbandi, 2016 [42]	Iran	25/23	30-37	8-10	1200-2000	5 days, three times in each day, 15 minutes in each time.
Lotfi, 2016 [43]	Iran	25/25	30-36	8-10	>1250	6 days, two times in each day, 15 minutes in each time.
Kenari, 2020 [44]	Iran	30/30	37-42	No. reported	>3000	3 days, once in each day, 15 minutes in each time.
Kianmehr, 2014 [45]	Iran	18/16	36-38	No. reported	2500-400	4 days, three times in each day, 15 minutes in each time.
Moghadam, 2012 [33]	Iran	16/18	37-42	No. reported	2500-400	2 days, three times in each day, 20 minutes in each time.
Nikzad, 2021 [46]	Iran	30/30	37-42	8-10	2500-4000	2 days, three times in each day, 15 minutes in each time.
Seyyedrasooli, 2014 [14]	Iran	25/18	37-42	No. reported	2500-400	4 days, once in each day, 15 minutes in each time.
Boskabadi, 2020 [47]	Iran	30/30	>30	<7	>2500	2 days, three times in each day, 20 minutes in each time.
Chen, 2011 [48]	Japan	20/22	37-41	8-10	2800-3600	5 days, two times in each day, 15-20 minutes in each time.
Dağ, 2019 [1]	Turkey	35/35	≥34	No. reported	≥1500	1 day, two times in each day, 15-20 minutes in each time.
Gözen, 2019 [49]	Turkey	44/46	37-41	8-10	2500-4000	2 days, three times in each day, 5 minutes in each time.
Korkmaz, 2020 [50]	Iran	27/29	37-42	8-10	2500-4000	1 day, two times in each day, 15 minutes in each time.
Lin, 2015 [13]	China	27/29	37-41	8-10	2500-3600	3 days, two times in each day, 15-20 minutes in each time.

## Data Availability

The data described in this article are openly available in the article. This data is also available in the text of articles that entered in this dose-response meta-analysis.
